# Molecular epidemiology of Hepatitis B virus genotypes in Pakistan

**DOI:** 10.1186/1471-2334-7-115

**Published:** 2007-10-08

**Authors:** Muhammad Masroor Alam, Sohail Zahoor Zaidi, Salman Akbar Malik, Shahzad Shaukat, Asif Naeem, Salmaan Sharif, Mehar Angez, Javed Aslam Butt

**Affiliations:** 1 Department of Virology, National Institute of Health, Islamabad, Pakistan; 2 Head of Department of Virology, Principal Investigator-WHO Regional Reference Laboratory for Polio Eradication Initiative, National Institute of Health, Islamabad, Pakistan; 3 Head of Department of Biochemistry, Quaid-i-azam University, Islamabad, Pakistan; 4 Head of Department of Gastroenterology, Pakistan Institute of Medical Sciences, Islamabad, Pakistan

## Abstract

**Background:**

Eight genotypes of Hepatitis B virus designated A-H, have been known but in Pakistan, no such data is available on the prevalent HBV genotypes. Therefore, the subject study was conducted to determine HBV genotypes in the indigenous Pakistani population.

**Methods:**

A total of 690 individuals were enrolled for HBV screening with EIA and nested PCR. Positive samples were further analyzed to determine HBV genotypes (A-F) by multiplex-PCR using type specific primers.

**Results:**

110 (15.94%) individuals were positive for HBV, including 64% males and 36% females. Out of these, 66 samples (65.34%) were classified into genotype D, 27 (26.73%) were of genotype B while 5(4.95%) had genotype A. In 3 (2.98%) samples, multiple genotypes were detected (genotype A+B; 2(1.99%) and genotypes B+D; 1(0.99%). Nine (8.18%) samples remained untyable.

**Conclusion:**

In Asia, genotypes B and C are the most prevalent but our study reveals that genotype D is predominant and HBV infection constitutes a significant health problem in Pakistan.

## Background

Hepatitis B virus (HBV) infection is a well recognized and major health problem leading to significant morbidity and mortality worldwide especially in the developing countries. Approximately, 2 billion people in the world have been infected by HBV [1], 400 million of who are chronic carriers [2]. The virus causes acute hepatitis of varying severity [3] and persists in 95% of children and 2–10% of adult patients [4] leading to chronic liver disease, cirrhosis, hepatocellular carcinoma [5] and even fulminant hepatitis [6]. In Pakistan, HBV infection rate is increasing day by day. The reason may be the lack of proper health facilities or poor economical status and less public awareness about the transmission of major communicable diseases like Hepatitis B virus, Hepatitis C virus and Human Immunodeficiency Virus.

Hepatitis B virus exhibits genetic variability with an estimated rate of 1.4 – 3.2 × 10^-5 ^nucleotide substitution per site per year [7] which resulted in well recognized subtypes of the virus. In addition, virus variants arise during replication as a result of nucleotide misincorporations in the absence of any proof reading capacity by the viral polymerase. HBV has been classified into 8 well defined genotypes on the basis of an inter-group divergence of 8% or more in the complete genomic sequence, each having distinct geographical distribution. Genotype A can be regarded as pandemic but is most commonly found in Northern Europe, North America and Central Africa, while genotype B predominates in Asia (China, Indonesia and Vietnam). Genotype C is found in the Far East in Korea, China, Japan and Vietnam as well as the Pacific rim and Island Countries, while genotype D, which is also more or less pandemic, is found in the Mediterranean countries, the Middle East extending to India, North America and parts of the Asia-Pacific region. Genotype E is related to Africa while genotype F is found predominately in South America, including among Amerindian populations, and also Polynesia. Genotype G has been found in North America and Europe while the most recently identified genotype H has been reported from America [8].

Since the discovery of HBV subtypes, their impact on the natural course of infection has been studied mainly in the South-East Asia where HBV is hyper endemic with prevailing genotypes B and C. The clinical significance of different HBV genotypes has become increasingly recognized in patients with acute and chronic infection. The course of HBV infection depends on several factors such as host genetic factors, age and genetic variability of the virus [9,10]. Genotype C induces a more severe disease, has higher scores for fibrosis and is more prevalent in cirrhotic patients as compared to genotype B [11]. Seroconversion from HBeAg to anti-HBeAg positivity occurs much earlier in genotype B than genotype C carriers [12]. Genotype C is found to have lower HBV DNA level than genotype A, B and D in the HBeAg positive patients [13]. Taken together, these studies suggest well established pathogenic, epidemiological, clinical and therapeutic differences among HBV genotypes. However, the epidemiology of mixed HBV genotype infections is very less understood [14].

In the present study, the molecular investigation of HBV genotypes was determined as there is no such data available in Pakistan. The infection markers were compared with patients' age; gender their clinical and molecular data to find out the possible correlation.

## Methods

Individuals are regularly screened for HBV infection at National Institute of Health (N.I.H) Islamabad which are referred from various hospitals and medical centers of the country. Subjects for the present study were selected randomly from the major and prestigious hospitals of each of the four provinces of the country. These hospitals also represent as a reference centre for the disease investigation. Because the current project is based on the reporting of HBV genotypes for the first time, therefore, no certain criteria like risk groups, age, gender was considered, however, samples positive for HCV and HDV were excluded from the study criteria.

A total of 690 individuals were included after approval from Research Committee of the Institute. Informed consent was obtained from all the individuals involved.

Serum HBsAg was assayed by Abbott-IMX HBsAg V2 Assay. DNA was extracted from 100 μl serum sample of 110 HBsAg positive patients using Biospin Blood Genomic DNA Mini-Prep Kit (Bioer Technology Co., Germany) according to the manufacturer's protocol, eluted in 70 μl buffer and stored at -20°C. Genotyping was performed using methodology as reported by Naito *et al., *2001 [15]. 50 μl reaction mixture was used containing 1× buffer, 20 μM primers, 2.4 μM dNTPs and 1.5 U Taq polymerase. 40 cycles was performed at 95°C for 1 min, 50°C for 30 sec, 72°C for 30 sec. The amplified product was run on 2.5% agarose gel and visualized under UV illuminator.

Data was analyzed using Epi-Info 6.0 [16]. A *p *value of less than or equal to 0.05 was considered as significant.

## Results

690 individuals were screened for the detection of hepatitis B surface antigen and HBV DNA. 110 (15.94%) were found positive for both infection markers [IMX HBsAg V2 Assay (EIA 3.0) and PCR]. Males were found to be more frequently infected as compared to females with a positivity ratio of 2.23: 1 respectively. Very young females of less than 20 years of age were not found to be positive for hepatitis surface antigen. 58 individuals positive for surface antigen were belonging to the age group ranging 21–40 years of age followed by the 39 positive individuals of 41–60 years age. Highest frequency of HBsAg positive males were found in the younger age with more than positive individuals falling under this 21–40 age group while very young and very old individuals were very less frequently infected by HBV. Majority of the females found to be infected with hepatitis B virus were aged more than 40 years. Infection was not found in very young females while only 3 were positive from post 60 years age (Table 1, 2).

**Table 1:** Age and Gender wise distribution of patients positive for Hepatitis B virus

**Age (years)**	**HBV positive Total**	**HBV + Male**	**HBV +ve Female**	**Mean ALT (U/L)**
1–20	06	06	0	275.5 ± 181.4
21–40	55	41	14	65.21 ± 35.33
41–60	39	22	17	57.54 ± 22.76
>60	10	07	03	59.85 ± 19.65
				
**Total**	**110**	**76**	**34**	

Mean ALT (U/L) is also shown.

**Table 2:** Geographical distribution of patients infected with Hepatitis B virus belonging to all the four provinces of the country

**Origin**	**Number of patients**	**HBsAg +ve**
**Punjab**	330	39
**Sindh**	105	25
**Balouchistan**	99	18
**NWFP**	156	28
		
**Total**	**690**	**110**

Out of the total 110 samples analyzed, 101 (91.81%) showed genotype specific bands while the remaining 9 HBV positive samples (8.19%) were found negative. 66 samples (65.34%) were classified into genotype D, 27 (26.73%) were of genotype B while 5(4.95%) had genotype A. In 3 (2.98%) samples, mix genotypes were detected as: [genotype A+D; 2(1.99%) and genotypes B+D; 1(0.99%)].

The prevalence of genotypes was assessed further with respect to patient's age (Table 3). There was no trend found in the distribution of genotypes among various age groups. However, genotype A was absent from individuals aged more than 60 years. Genotype B and D were present in all age groups while the patients infected with multiple genotypes were all belonging to age group between 41–60 years. This relationship was, however, found non significant (*p *= 0.142).

**Table 3:** Origin based distribution of Hepatitis B virus genotypes among Hepatitis B virus infected individuals belonging to various age groups

	**Number of Individuals**		
**Origin Age Group**	**Genotype A**	**Genotype B**	**Genotype D**

**Punjab (30)**			
1–20	x	2	x
21–40	2	4	11
41–60	x	3	8
>60	x	x	x
**Sindh (25)**			
1–20	x	1	x
21–40	1	1	6
41–60	1	x	12
>60	x	2	1
**Balouchistan (18)**			
1–20	1	1	1
21–40	x	1	5
41–60	x	2	3
>60	x	x	4
**NWFP (28)**			
1–20	x	x	x
21–40	x	4	8
41–60*	x	4	6
>60	x	2	1
			
**Total**	**5**	**27**	**66**

* represents the age group to which belong the three individuals infected with multiple HBV genotypes.

**Figure 1:**
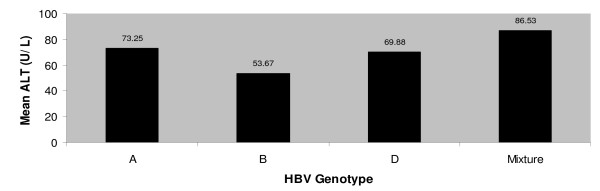
Mean ALT (U/L) of patients infected with different genotypes of hepatitis B virus.

The results of alanine aminotransferase level testing were compared among the individuals infected with different genotypes. There was no significant relationship found among individuals however, the highest ALT level (86.53 ± 20.67) was found in the individuals infected with more than one genotype. It was found that genotype A and genotype D positive individuals showed two fold increase in ALT level with 73.25 ± 31.23 U/L and 69.88 ± 33.12 U/L respectively. The ALT level of genotype A infected individuals was the lowest of all other patients with mean value of 53.67 ± 19.87 U/L (Figure 1).

## Discussion

Hepatitis B virus (HBV) infection is a global health problem with its continuously increasing burden in the developing countries like Pakistan. About 400 million people of the world population are chronic carriers of the virus [17]. In addition to serological classification of HBV isolates into nine subtypes on the basis of HBsAg determinants [18], a genetic classification based on the comparison of complete genomes has defined 8 genotypes of HBV (A to H). To investigate the epidemiological distribution of HBV genotypes in our local population, we applied a multiplex PCR based methodology. Although the most common method for HBV genotyping is by PCR-RFLP technique, but it is reported that HBV genotyping by multiplex PCR is more sensitive than genotyping system using RFLP analysis. Leblebicioglu reported that nested PCR methodology for HBV genotyping is 1000-fold more sensitive than PCR-RFLP [19]. Lim also deduced that HBV genotyping method using nested PCR with type specific primers is more sensitive than PCR-RFLP [20].

Total screening of 690 individuals resulted in 110 infected patients with hepatitis B virus representing 15.94% of the total subjects. This represents quite a higher number of infected individuals. According to WHO, Pakistan falls under the endemic region with 3% HBV infected country population. Different studies have been conducted in the country to assess the disease burden in the indigenous population. All such studies present a varying rate of infection based on the study design, population selected, diagnostic assays and demographical and epidemiological variation. According to various study groups, the HBV prevalence rate has been reported as 2–10% among healthy blood donors; 5–9% among health care personnel; 3.6–18.66% among the general population; 3.16% among the pregnant women; 10–20% in patients with provisional diagnosis of hepatitis and 3.16–10.4% among professional blood donors [21]. We have found 15.94% infected population out of the study subjects because almost all of the individuals included in the current study were clinically infected patients who were referred to hospitals and medical centers for their medication and physicians advise. This figure, however, does not represent the overall picture of the total country's population prevalence rate infected with hepatitis B virus.

The current study reveals that males are more frequently positive for HBV infection as compared to females. The prevalence of HBV infection was found to be higher in males (69.1%) than females (30.9%). Also, HBV infection is found to be significantly higher in persons with age between 21–40 years. Leung also reported that men aged 40 years or old were most affected HBV patients as compared to females [22]. The reason may be that the men are more exposed to the risk factors. In Pakistan, males are more educated and employed working outsides their homes or in agricultural lands while women are mostly involved in house activities based on the cultural and religious preferences and influences. Therefore, it is well understood to analyze the difference of frequency of infection among the gender category. Males are more commonly involved in the trends and practices leading to the transmission of infections like blood transfusions and barber visiting so becoming more prone to get the infection at a much frequent rate as compared to females. The vertical transmission of HBV infection in Pakistan has been reported to be very low rendering the horizontal transmission a major risk factor for the infection acquisition. It has already been documented that the HBsAg prevalence rate in pregnant women was 2.5% in Pakistan, out of which 17% and 61% were HBeAg and anti-HBeAg positive thus indicating the vertical transmission a less important cause of HBV transmission [23].

The 110 samples analyzed for genotyping indicated the highest prevalence of genotype D followed by genotype B, A and co-infection with multiple genotypes including HBV/A+D and HBV/B+D. Genotypes C, E and F were not found in any patient indicating absence of these strains in this region. Apart from Pakistan, the initial studies on HBV genotyping in the other regions of Asia revealed that genotypes B and C are the most prevalent genotypes in this region. It was because of the fact that all such studies were reported mostly from Japan and China where genotypes B and C are the most prevalent. Later on, it was found that all the seven HBV genotypes can be found in Asia [24]. For instance, the predominant genotypes in India are Genotype A and D [25]; the predominant HBV genotype in Afghanistan was found to be genotype D [26]. The epidemiological data about HBV genotypes in various Asian countries revealed the presence of all seven genotypes in Asia, particularly the pre-dominance of genotype D.

It is of much important finding that we have reported such patients infected with multiple (more than one) HBV genotypes for the first time in Pakistan. This is in accordance with a number of very recent studies from different regions of the world. Hannoun found 8% of HBV patients with genotype mixture [27]. Toan found that chronic patients are more prone to be infected with more than one HBV genotype than acutely infected patients [19]. Genotypes mixture in HBV patients is also common in Thailand [28]. 16% HBV cases were positive for HBV genotype mixture in France [29].

In our study, 9 HBV DNA positive samples remained negative for HBV genotypes. It may be assumed that such samples represent recombinant or new genotypic variants present in our population that can be resolved after sequencing and further analysis. Because, some minor HBV genotypes as well as novel or distinct genotypic groups may be present in any population besides major genotypes [30].

## Conclusion

HBV genotypes influence the severity of liver disease and response to interferon and antiviral therapy. These are also expected to influence the emergence of resistant. Patients infected with HBV of certain genotypes can be directed to the other therapeutic options to spare the cost and burden of treatment [31]. Therefore, the appropriate and definite knowledge of all the HBV genotypes prevalent in a certain region is of immense importance for the proper and effective management of HBV patients.

As we have reported for the first time the prevalence of HBV genotype D as the pre-dominant genotype in Pakistan and also the patients infected with more than one HBV genotypes, therefore, it is of worth important consideration for clinicians to adopt better strategies for appropriate prevention and cure of infection. Furthermore, such studies are important for epidemiological reasons as well as developing effective therapeutic management against such infections.

## Competing interests

The author(s) declare that they have no competing interests.

## Authors' contributions

SSZ and SAM designed the Research project and gave a critical view of manuscript writing. JAB helped in collecting the samples. SS collected the epidemiological data. AN analyzed the data statistically. SS and MA performed the serological assays. MMA and SS performed the molecular assays and wrote the manuscript. All the authors have read and approved the final manuscript.

## Pre-publication history

The pre-publication history for this paper can be accessed here:



## References

[B1] Zuckerman JN, Zuckerman AJ (2000). Current topics in hepatitis B. Journal of infections.

[B2] Lee WM (1997). Hepatitis B infection. New England Journal of Medicine.

[B3] Heerman KH, Gerlich WH, Michael C, Schaefer S, Thomssen R (1999). Quantitative detection of heopatitis B virus DNA in two international reference plasma preparartions. Journal of Clinical Microbiology.

[B4] Bowyer SM, Sim GM (2000). Relationship within and between the genotypes of Hepatitis B virus at point across the genome: footprints of recombination in certain isolates. Journal of General Virology.

[B5] Abe A, Kazuaki I, Take AT, Junko K, Nooki K, Satoshi T, Mkoto Y, Michinori K (1999). Quantification of Hepatitis B virus genomic DNA by Real-Time detection. Journal of Clinical Microbiology.

[B6] Mahoney FJ (1999). Update on diagnosis, management, and prevention of hepatitis B virus infection. Clinical Microbiology Reviews.

[B7] Orito E, Mizokami M, Ina Y, Moriyama EN, Kameshima N, Yamamoto M, Gojobori T (1989). Host dependent evolution and a genetic classification of the hepadnavirus family based on nucleotide sequence. Proceedings of National Academy of Sciences USA.

[B8] Kramvis A, Kew M, Francois G (2005). Hepatitis B virus genotypes. Vaccine.

[B9] Gunther S, Fischer L, Pult I, Sterneck M, Will H (1999). Naturally occurring variants of hepatitis B virus. Advances in Virus Research.

[B10] Hunt CM, McGill JM, Allen MI, Condreay LD (2000). Clinical relevance of hepatitis B viral mutations. Hepatology.

[B11] Kao JH, Chen PJ, Lai MY, Chen DS (2000). Hepatitis B genotypes correlate with clinical outcomes in patients with chronic hepatitis B. Gastroenterology.

[B12] Sumi H, Yokosuka O, Seki N (2003). Influence of Hepatitis B virus genotypes on the progression of chronic type B liver disease. Hepatology.

[B13] Westland C, Delaney W, Yang H (2003). Hepatitis B virus genotypes and virologic response in 694 patients in phase III studies of adefovir dipivoxil1. Gastroenterology.

[B14] Chen BF, Chen PJ, Jow GM, Sablon E, Liu CJ, Chen DS, Kao JH (2004). High prevalence of mixed genotype infections in hepatitis B virus infected intravenous drug users. J Med Virol.

[B15] Naito H, Hayashi S, Abe K (2001). Rapid and specific genotyping system for hepatitis B virus corresponding to six major genotypes by PCR using type specific primers. Journal of Clinical Microbiology.

[B16] EPI-INFO A data analysis software developed by the Centers for Disease Control and Prevention (Atlanta, GA).

[B17] Lee WM (1997). Hepatitis B infection. New England Journal of Medicine.

[B18] Courouce-Pauty AM, Lemaire JM, Roux JF (1978). New Hepatitis B surface antigen subtypes inside the *ad *category. Vox Sang.

[B19] Leblebicioglu H, Eroglu C, members of the hepatitis study group (2004). Acute hepatitis B infection in Turkey: Epidemiology and genotype distribution. Clinical Microbiology and Infection.

[B20] Lim CK, Tan MTJ, Khoo SBJ, Ravichandran A, Low MH, Chan CY, Ton HS (2006). Correlations of HBV genotypes, mutations affecting HBeAg expression and HBsAg/anti-HBe status in HBV carriers. International Journal of Medical Sciences.

[B21] (2002). HBV and HCV review article. JCPSP.

[B22] Leung N (2002). Treatment of Chronic hepatitis B : Case selection and duration of therapy. Journal of Gastroenterology and Hepatology.

[B23] Abbas Z, Jafri W, Shah SH, Khokhar N, Zuberi SJ (2004). Pakistan Society of Gastroenterology. PSG consensus statement on management of hepatitis B virus infection-2003. J Pak Med Assoc.

[B24] Toan NL, Song LH, Kremsner PG, Duy DN, Binh VQ, Koeberlein B, Kaiser S, Kandolf R, Torresi J, Bock CT (2006). Impact of Hepatitis B virus genotypes and genotype mixtures on the course of liver disease in Vietnam. Hepatology.

[B25] Thakur V, Guptan RC, Kazim SN, Malhotra V, Sarin SK (2002). Profile, spectrum and significance of HBV genotypes in chronic liver disease patients in the Indian subcontinent. Journal of Gastroenterology and Hepatology.

[B26] Amini-Bavil-Olyaee S, Alavian SM, Adeli A, Sarrami-Forooshani R, Sabahi F (2006). Hepatitis B virus genotyping, core promoter and precore/core mutations among afghan patients infected with hepatitis B: a preliminary report. Journal of Medical Virology.

[B27] Hannoun C, Krogsgaard K, Horal P, Lindh M (2002). Genotype mixtures of hepatitis B virus in patients treated with interferon. Journal of Infectious Diseases.

[B28] Jutavijittum P, Jiviriyawat Y, Yousukh A, Kunachiwa W, ToriYama K (2006). Genotypes of Hepatitis B virus among voluntary blood donors in northern Thailand. Hepatology Research.

[B29] Halfon P, Bourliere M, Pol S, Benhamou Y, Ouzan D (2006). Multicenter study of Hepatitis B virus genotypes in France: Correlation with liver fibrosis and hepatitis B e antigen status. Journal of Viral Hepatology.

[B30] Bowyer SM, Sim GM (2000). Relationship within and between the genotypes of Hepatitis B virus at point across the genome: footprints of recombination in certain isolates. Journal of General Virology.

[B31] Akuta N, Kumada H (2005). Influence of hepatitis B virus genotypes on the response to antiviral therapies. Journal of Antimicrobial Chemotherapy.

